# Surgical outcomes of 23-gauge transconjunctival pars plana vitrectomy combined with lensectomy for glaucomatous eyes with extremely shallow anterior chamber and cataract

**DOI:** 10.1186/s12886-015-0179-8

**Published:** 2016-01-04

**Authors:** Zhaotian Zhang, Shaochong Zhang, Xintong Jiang, Suo Qiu, Yantao Wei

**Affiliations:** State Key Laboratory of Ophthalmology, Zhongshan Ophthalmic Center, Sun Yat-sen University, No.54, South Xianlie Road, Guangzhou, 510060 China

**Keywords:** 23 gauge, Transconjunctival, Pars plana vitrectomy, Pars plana lensectomy, Glaucoma, Shallow anterior chamber, Cataract

## Abstract

**Background:**

Glaucoma combined with an extremely shallow anterior chamber and cataracts remains as a complex condition to deal with. And the emergence of microincision vitrectomy surgery (MIVS) system may provide an ideal option for the treatment of that. We report a clinical study of surgical outcomes of 23-gauge transconjunctival pars plana vitrectomy (PPV) combined with lensectomy in the treatment of glaucomatous eyes with extremely shallow anterior chamber and cataract.

**Methods:**

Prospective, nonrandomized and noncomparative case series study. Consecutive patients with secondary glaucoma, extremely shallow anterior chamber and cataract were recruited to have combined surgeries of 23-gauge transconjunctival pars plana vitrectomy and lensectomy. The main outcomes were best corrected visual acuity (BCVA), intraocular pressure (IOP), anterior chamber depth (ACD), number of anti-glaucoma medications and surgery-associated complications.

**Results:**

Seventeen consecutive patients with secondary glaucoma, extremely shallow anterior chamber and cataract were recruited. The mean follow-up was 21.2 ± 8.8 months. Postoperatively, there was no significant improvement of BCVA (*P* = 0.25). The mean intraocular (IOP) decreased significantly from 43.14 ± 6.53 mmHg to 17.29 ± 1.80 mmHg (*P* < 0.001), and the mean depth of anterior chamber increased significantly from 0.507 ± 0.212 mm to 3.080 ± 0.313 mm (*P* < 0.001). The mean number of anti-glaucoma medications decreased from 4.1 ± 0.8 to 0.6 ± 0.8 (*P* < 0.001). No severe vision-threatening intra- or post-operative complications occurred.

**Conclusions:**

Glaucoma with an extremely shallow anterior chamber and cataract can be managed well with the combined surgeries of 23-gauge pars plana vitrectomy and lensectomy. The surgical procedure is an effective and safe method to resolve the pupillary block and deepen the anterior chamber.

## Background

Glaucoma combined with an extremely shallow anterior chamber and cataracts is a complex condition often associated with malignant glaucoma, phacomorphic glaucoma, spherophakia or nanophthalmos. In such patients, the primary mechanism is anterior dislocation of lens-iris diaphragm resulted from pupillary or cilliary blockage [1–5]. Elevated intraocular pressure (IOP) and long-lasting contact of the lens-iris diaphragm to the corneal endothelium eventually lead to corneal decompensation and severe impairment of visual function. Therefore, besides control of the IOP, cataract extraction and anterior chamber formation are considered to be necessary [1, 5]. However, the crowded anterior chamber poses several difficulties during phacoemulsification, including poor construction of corneal incision, more endothelial cell loss, iris prolapse, difficult capsulorhexis and high risk of complications [6, 7].

Previous studies have demonstrated the effectiveness of vitrectomy combined with lensectomy or phacoemulsification in the treatment of malignant glaucoma [6, 8]. However, conventional 20-gauge vitrectomy system requires opening of the conjunctiva, which causes extensive conjunctival scarring [9, 10]. With the rapid development of surgical instrumentations, the microincision vitrectomy surgery (MIVS) system provides a promising technique with multiple advantages over the 20-gauge vitrectomy system [11–15]. MIVS system (23-gauge or 25-gauge) may be a feasible alternative to overcome the technical difficulties mentioned above [8–11].

According to the latest literatures, there have been few studies on the use of 23 or 25-gauge MIVS in the severe anterior segment eyes diseases. Therefore we conducted the current study to investigate its potential application in this series of diseases. In the study, we adopted 23-gauge transconjunctival PPV combined with pars plana lensectomy (PPL) in eyes with glaucoma, cataract and extremely shallow anterior chamber. Considering the severe irreversible vision defect induced by glaucoma and possible complications after intraocular lens (IOL) implantation, IOL was not determined to be implanted in any eyes of the patients. The aim of the surgery is to remove the lens and vitreous, create a communication between the anterior chamber and the vitreous cavity, and thereby reform the anterior chamber.

## Methods

The study was approved by the Institutional Review Board of Zhongshan Ophthalmic Center affiliated to Sun Yat-sen Univiersity (Guangzhou, China). And it was performed in accordance with the World Medical Association’s Declaration of Helsinki. Inclusion criteria for patients included medically uncontrolled IOP, extremely shallow central and peripheral anterior chamber and lens nucleus of grade 2or 3. Exclusion criteria were patients with uncontrolled ocular infection, significant opacity of the cornea, lens nucleus of grade 4 or harder, severe systemic diseases and those unable to have scheduled follow-ups. The procedures were fully explained to each patient, and written informed consent was obtained from each subject. Written consent of publishing case details was also obtained from each subject. Seventeen eyes of 17 consecutive patients were prospectively recruited from January 2011 to February 2013 at Zhongshan Ophthalmic Center of Sun Yat-sen University.

All eligible patients underwent comprehensive ophthalmologic examinations, including best-corrected visual acuity (BCVA), non-contact tonometry and slit-lamp microscope. The nuclear sclerosis was graded using the Lens Opacity Classification System (LOCS III) [16]. The anterior chamber angle and anterior chamber depth (ACD) were examined by ultrasound biomicroscope (UBM Plus, Model P45; Pardigm Medical Industries, Salt Lake City, UT, USA). The ACD was defined as the distance between the posterior corneal surface and the anterior lens surface. B-scan ultrasonography was performed to determine status of the vitreous and retina. The axial length was also measured by A-scan ultrasound biometry.

One hour before surgery, 20 % mannitol was intravenously administered to all the patients. All surgeries were performed under retrobulbar anesthesia by one experienced ophthalmologist (S.Z) using 23-gauge system (Constellation Vitrectomy System, Alcon Laboratories, USA). Transconjunctival incisions were created about 3.0–3.5 mm from the limbus using trocars at the inferotemporal, superotemporal, and superonasal quadrants. Using the 23-gauge instruments, the anterior vitreous was firstly cut off. And then lensectomy was performed to extract the lens nucleus and cortex. Peripheral lens capsule was preserved for the secondary implantation of posterior IOL. Once there was any nucleus fragment dropping into the vitreous cavity, total vitrectomy was determined to remove the lens fragments and the residual vitreous. By means of the previous steps, the communication between the anterior chamber and the vitreous cavity was constructed. The anterior chamber was accessed through the superior trocar and the pupil, viscoelastic agents were then injected to separate the peripheral iris-corneal adhesion. As mentioned above, we determined that IOL implantation would not a feasible option for the patients, so all the eyes were not implanted with IOL finally. After all the procedures were completed, the cannulas were removed. And the incisions were sealed by a single stich with 7–0 Vicryl sutures if obvious leakage was observed.

Postoperatively, tobramycin, nonsteroidal anti-inflammatory and dexamethasone eye drops were consecutively administered four times daily for 4–6 weeks. Anti-glaucoma eye drops or oral medicine was administered if necessary.

Follow up examinations were scheduled at 1 week, 1 month, 3 months, 6 months, and 1 year after the surgery. If necessary, extra revisits were performed. At each postoperative visit, BCVA was assessed with Snellen visual charts, IOP was measured by non-contact tonometry, slit-lamp microscope and ophthalmoscope were performed, and the number of anti-glaucoma medications being used was also recorded. All the postoperative ACD was examined by anterior segment coherence tomography (AS-OCT, VisanteTM; Carl Zeiss Meditec, Dublin, CA, USA). In the aphakic eyes, the ACD was defined as the distance between the posterior corneal surface and the plane of the pupil. Improved VA was defined as improvement of more than one Snellen line, same as within one line and worse as loss of more than one Snellen line.

All Snellen visual acuity values were converted to the logarithm of the minimum angle of resolution (logMAR) for statistical analysis. Visual acuity of light perception (LP) was assigned as 2.9 logMAR, hand movements (HM) as 2.6 logMAR, and counting fingers (CF) as 2.3 logMAR. All data were analyzed using the SPSS 13.0 statistical software (SPSS Inc., Chicago, IL, USA). The Shapiro normality test was performed on all the continuous data. Paired t-test and Wilcoxon matched-pairs signed rank test were used as appropriate. All the continuous data were expressed as mean ± standard deviation (SD). A *P* value less than 0.05 was considered statistically significant.

## Results

**Fig. 1:**
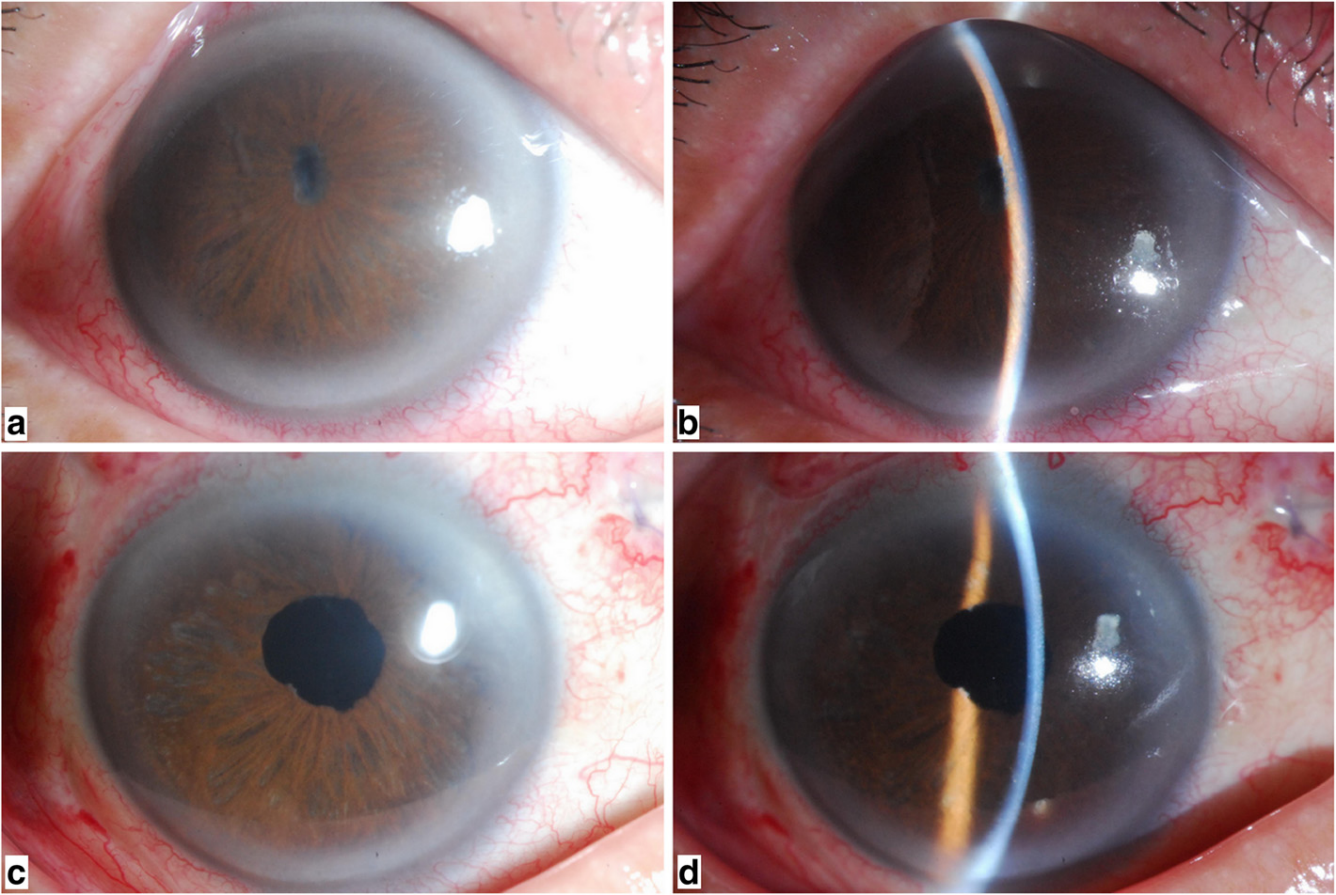
A 45-year-old man presented with glaucoma secondary to uveitis in his right eye (Case 9). Before surgery: **a** anterior segment photograph shows marked iris atrophy and pupillary occlusion. **b** slit lamp biomicroscopy examination revealed an extremely shallow anterior chamber. The central ACD was 0.261 mm. One week after surgery: **c** The image on the left demonstrates a quiet anterior chamber and a relative normal pupil. **d** The slit lamp photography shows a significantly deep anterior chamber. The central ACD has increased to 3.069 mm at the center after 23-gauge PPV and PPL

There were totally 17 consecutive patients (17 eyes) included in the study, with 10 eyes (58.8 %) diagnosed as malignant glaucoma, three eyes (17.6 %) as phacomorphic glaucoma, two eyes (11.8 %) as glaucoma secondary to spherophakia and two eyes (11.8 %) as glaucoma secondary to uveitis. The mean age of the patients was 56.35 years (range 40–71 years). Previous surgical interventions included trabeculectomy in ten eyes, anterior chamber reformation in five eyes, and laser peripheral iridectomy in two eyes. The lens nucleus density was classified from grade 2 in seven eyes (41.2 %) to grade 3 in seven eyes (41.2 %) and mature white cataract was diagnosed in three eyes (17.6 %). The mean axial length was 22.31 ± 0.94 mm (range 21.23–24.83 mm). The mean postoperative follow-up duration was 21.2 ± 8.8 months (range 9–40 months). The baseline characteristics of all patients were summarized in Table 1. A typical case (Case 9) was demonstrated in Fig. 1.

**Table 1:** The characteristics of 17 patients with secondary glaucoma, extremely shallow anterior chamber and cataract

No	Age	Sex	Eye	Diagnosis	Nucleus density	Prior surgeries	BCVA		IOP (mmHg)		No. of glaucoma medication		ACD (mm)		AL (mm)	Follow-up (month)
							Pre-	Post-	Pre-	Post-	Pre-	Post-	Pre-	Post-		
1	65	F	os	Phaco glaucoma	Mature white	No	HM	HM	49.4	16.5	4	0	0.544	2.869	22.77	18
2	41	F	os	Malig glaucoma	2	Trab+ACF	0.02	0.02	39.5	18.7	4	0	0.317	2.955	21.23	12
3	69	M	os	Malig glaucoma	3	Trab	0.04	0.05	43.2	18.5	5	1	0.405	2.797	22.27	30
4	71	M	os	Phaco glaucoma	Mature white	No	HM	HM	41.7	17.4	5	0	0.220	2.980	24.83	24
5	59	F	os	Malig glaucoma	2	Trab+ACF	HM	FC	42.0	18.9	4	1	0.693	2.750	22.21	30
6	63	F	od	Malig glaucoma	3	Trab+ACF	0.04	0.02	38.5	18.2	3	0	1.050	3.463	22.30	36
7	67	M	os	Malig glaucoma	3	Trab	HM	HM	49.8	17.5	5	2	0.762	2.676	23.85	18
8	68	F	os	Spherphakia, glaucoma	2	No	FC	0.02	35.3	17.3	3	0	0.580	3.581	21.60	24
9	45	F	od	Uveitis, glaucoma	3	LPI	LP	FC	41.4	14.7	4	0	0.261	3.069	23.49	30
10	58	F	od	Malig glaucoma	3	Trab	FC	0.02	50.9	16.2	5	1	0.630	2.750	21.72	18
11	42	F	od	Uveitis, glaucoma	3	LPI	HM	FC	33.6	16.1	4	0	0.378	2.823	21.62	24
12	40	F	od	Malig glaucoma	2	Trab	HM	HM	37.5	15.4	3	0	0.542	2.951	21.94	12
13	62	M	od	Phaco glaucoma	Mature white	No	HM	FC	42.8	20.8	3	1	0.460	3.520	22.31	36
14	57	M	os	Malig glaucoma	2	Trab+ACF	HM	FC	60.3	18.5	5	2	0.635	2.962	21.98	12
15	44	F	od	Spherphakia, glaucoma	2	no	0.02	0.02	39.4	15.7	4	0	0.322	3.551	21.67	9
16	50	F	os	Malig glaucoma	2	Trab+ACF	FC	FC	42.7	14.1	5	1	0.524	3.360	22.10	15
17	57	M	os	Malig glaucoma	3	Trab	HM	FC	45.3	19.5	4	2	0.295	3.298	21.45	12

*BCVA* best-corrected visual acuity; *CF* counting fingers; *HM* hand motion; *M* male; *F* female; *ACD* anterior chamber depth; *AL* axial length; *IOP* intraocular pressure; *Trab* trabeculectomy; *LPI* laser peripheral iridectomy; *ACF* anterior chamber reformation; Phaco glaucoma: phacomorphic glaucoma; Malig glaucoma: malignant glaucoma; Pre-: preoperative; Post-: postoperative

Preoperatively, the BCVA was 8/200 in two eyes (11.8 %), 4/200 in two eyes (11.8 %), CF in three eyes (17.6 %), HM in nine eyes (52.9 %), and LP in one eye (5.9 %). At the final visit, the BCVA was 10/200 in one eye (5.9 %), 4/200 in five eyes (29.4 %), CF in seven eyes (41.2 %), HM in four eyes (23.5.8 %). The preoperative and postoperative logMAR BCVA had no statistical difference (2.32 ± 0.47 VS 2.28 ± 0.42) (*P* = 0.25).

**Fig. 2:**
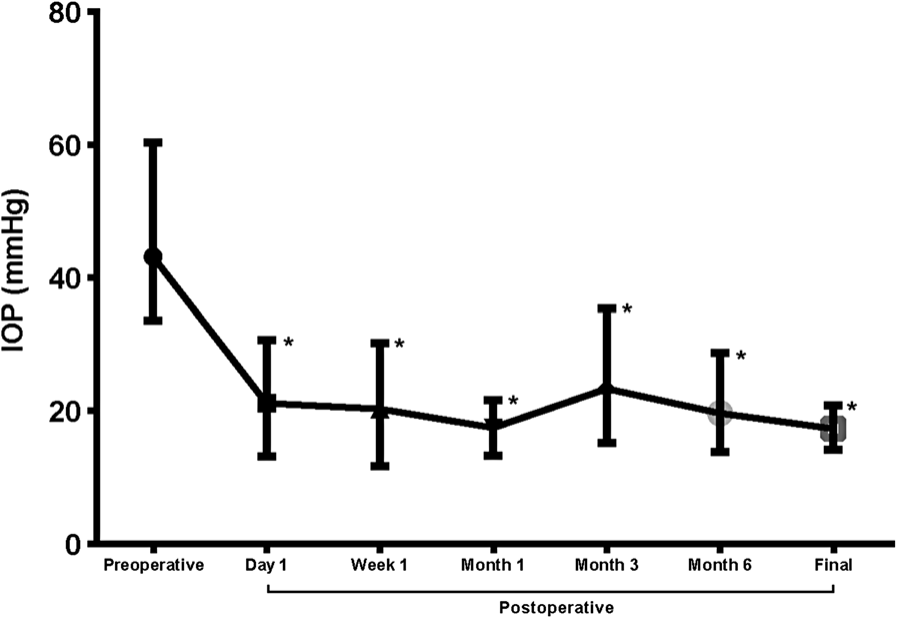
IOP changes before and after 23-gauge PPV and PPL (* = presence of statistical significance to the preoperative IOP)

The mean IOP was 43.14 ± 6.53 mmHg (range 33.6–60.3 mmHg) preoperatively, which significantly decreased to 21.12 ± 4.76 mmHg (*P* < 0.001) at 1 day and 20.28 ± 4.85 mmHg (*P* < 0.001) at 7 days postoperatively. One month after the surgery, the mean IOP of 17.45 ± 2.42 mmHg was maintained with/without topical anti-glaucoma medications. However, a slow elevation of IOP was observed in two eyes during the follow-up (Case 9 and Case 11), which underwent glaucoma valve implantation due to the failure of IOP-lowering medications. At the final visit, the mean IOP was 17.29 ± 1.80 mmHg (range 14.4–20.8 mmHg) in 17 eyes. Changes in IOP measurements during the follow-up were shown in Fig. 2.

With regard to anti-glaucoma medications, patients were administered a mean of 4.1 ± 0.8 (range 3–5) medications before surgery. At the last follow-up, anti-glaucoma medications had decreased significantly to 0.6 ± 0.8 (range 0–2) (*P* < 0.001). And nine eyes (52.9 %) maintained the IOP in a normal range without any anti-glaucoma eye drops.

The mean preoperative ACD was 0.507 ± 0.212 mm (range 0.220–1.05 mm). At the final follow-up, the mean ACD was 3.080 ± 0.313 mm (range 2.676–3.581 mm), which was statistically significant compared with the preoperative one (*P* < 0.001).

There were no severe perioperative complications occurred. During the surgery, slight iris hemorrhage occurred in one eye (Case 9) during the separation of iris posterior synechia, but it stopped soon after temporarily rising of irrigation pressure. Peripheral retinal hole was found in one eye, which subsequently received retinal laser photocoagulation. At the end of the surgery, four eyes (23.5 %) required suturing to stop visible leakage at the sclerotomy sites. No postoperative complications such as hypotony, conjunctival leakage, corneal decompensation, endophthalmitis, suprachoroidal hemorrhage, vitreous hemorrhage, choroidial detachment, or retinal detachment were observed. Failure of pre-existing filtering bleb was not found in any eyes.

## Discussion

Although this study enrolled patients with different types of glaucoma, the common features of all were medically uncontrolled IOP (43.14 ± 6.53 mmHg) and extremely shallow anterior chamber (0.507 ± 0.212 mm). In our case series, lens extraction was considered to be the definite treatment to deepen the anterior chamber and relieve pupillary block. However, the crowded anterior chamber disturbs the surgical manipulation of phacoemulsification in such eyes. The peripheral iridocorneal apposition makes it difficult to properly construct a clear corneal incision. The narrow anterior chamber puts the cornea under higher risk of damage by ultrasound waves and/or mechanical contact of the surgical instruments. Additionally, corneal edema and pupillary abnormities are commonly found in this kind of patients, both of which may increase the difficulty of capsulorhexis [6, 7]. Consequently, clear corneal phacoemulsification was considered to be fraught with higher risk of intra- and postoperative complications in our case series.

In such instance, PPV combined with PPL may be a relatively safer manipulation. Using vitrectomy cutter, cataract can be managed with enough working space from vitreous cavity. The lens was extracted by mechanical cutting, which exert less damage to the corneal endothelial cells as compared to ultrasound energy in phacoemulsification. Moreover, there was less need of repeated exchange of instruments, which would reduce IOP fluctuation during the procedure. In the current study, 23-gauge vitrectomy instrumentations were adopted to remove cataract and anterior vitreous through the transconjunctival incision. The main advantage of 23-gauge system is that incision of the conjunctiva is not required, which reduces operating times, minimizes conjunctiva trauma, and decreases postoperative inflammation [12–15]. Considering the rigidity and effectiveness of its instrumentations, 25-gauge system was not used in the present study.

According to our data, the combined surgery was effective in relieving the pupil block and reforming the anterior chamber. Intraoperatively, we observed significant deepening of the anterior chamber and elimination of aqueous blockade in all eyes. At the final revisit, the anterior chamber was maintained well with a mean depth of 3.080 mm. In our case series, the majority of patients (58.8 %) were diagnosed as malignant glaucoma. Previous studies have reported that PPV is an effective treatment to break the cycle of aqueous misdirection. However, several reports showed that the recurrence of malignant glaucoma is more likely in phakic eyes undergone PPV alone (26 ~ 75 %) compared with pseudophakic eyes and those received PPV and PPL (0 ~ 17 %) [8–10]. For these reasons, it is concluded that vitrectomy combined with lens extraction provide therapeutic advantage in phakic eyes with malignant glaucoma. In our surgical procedure with 23-gauge vitrectomy system, PPV and PPL were performed via the same transconjunctival sclerotomies without the creation of corneal incision. During the mean follow-up of 21.2 months, none of the patients suffered a recurrence of aqueous misdirection.

Although the anterior chamber was effectively deepened in all eyes, the aqueous outflow route was not successfully reconstructed in some cases due to the extensive peripheral anterior synechia or irreversible trabecular injury. Despite an initial normalizing of the IOP with/without anti-glaucoma medications in all patients, we observed a slow increase of IOP in two eyes with initial diagnosis of uveitis. During the follow-up period, implantations of Ahmed valve drainage device (New World Medical, Rancho Cucamonga, CA, USA) were eventually performed in these eyes (Case 9 and Case 11). Un Chul Park et al. reported that the success rate of Ahmed valve implantation in the vitrectomized group was similar to that of the nonvitrectomized group [17]. Nevertheless, conventional 20-gauge vitrectomy has been reported to cause conjunctiva scarring and recession in multiple quadrant, which may induce impairment of the pre-existing filtering bleb or exert challenge to the further glaucoma surgery [18]. In the current study, we found that the conjunctival adhesion caused by 23-gauge trocar was minimal, which didn’t disturb the implantation of the drainage device. Moreover, there was no suturing-associated failure of pre-existing filering bleb observed in our cases series. At the end of the follow-up, the mean IOP was 17.29 ± 1.80 mmHg with no need of glaucoma medications in 52.9 % of eyes.

The major complication of MISV is postoperative hypotony (IOP < 5 mmHg) resulted from leaking sclerotomies. As previously reported, the incidence of postoperative hypotony was 2.6 % after 23-gauge vitrectomy and 3.8 to 20 % after 25-gauge MIVS. [19–22] Postoperative hypotony can precipitate the occurrence of delayed subchoroidal hemorrhage, the incidence of which is 10-fold greater than that in the intraoperative period [23, 24]. In the present study, postoperative hypotony was not found in any of the case. The mean IOP was 21.12 ± 4.76 mmHg at 1 day and 20.28 ± 4.85 mmHg at 7 days after the surgery. The meticulous checks for the leaking sclerotomy and appropriate suturing were considered as the main causes of the low incidence of postoperative hypotony in our case series. The rate of sclerotomy suturing in this study was 23 %, which was higher than that reported by the available literatures (1.3–11.2 %) for 23-gauge vitrectomy [20, 25, 26]. The possible explanation was that vitreous base dissection was performed more thoroughly in most cases, which resulted in poorer closure due to the reduced “plugging effect” of remnant vitreous under the sites of sclerotomy. The sclerotomies were sutured by single-needle stitch to minimize the scar formation of corresponding conjunctiva. In our case series, the stable postoperative IOP may explain the low rate of complications including suprachoroidal hemorrhage, vitreous hemorrhage, choroidial detachment, and retinal detachment.

As described by Liu et al., phacoemulsification-posterior capsulorhexis–anterior vitrectomy– intraocular lens (IOL) implantion was performed in patients with phakic malignant glaucoma, which helped to improve the postoperative visual acuity [27]. Howerver, according to our data, the preoperative visual acuity was quite poor (2.32 ± 0.47) and 88.2 % eyes had BCVA of 20/400 or worse. The main causes of reduced vision in our patients were corneal edema, cataract, and glaucomatous optic nerve damage. Even after removal of lens, no significant increase in the mean BCVA was observed at the end of the follow-up. Owing to the little benefit of visual function achieved from the surgery, the goal of our surgery is to reform the anterior chamber, decrease the IOP and preserve existing vision. Therefore, the IOL was not determined to be implanted in our cases, which might reduce the surgery manipulation and the risks of complications.

Despite the advantages mentioned above, our study also has some limitations. One drawback was that ultrasonic fragmatome is not available in the 23-gauge vitrectomy system. It is almost impossible to manipulate the lens nucleus more than grade 4 using 23-gauge vitrectomy cutter. For this reason, eyes with lens nucleus of grade 4 or harder was considered as contraindication for this microincision technique, and thus not included in this study. This limitation may be resolved by the advancement in surgical instrumentation. More recently, Spierer et al. describe the application of a 20-gauge transconjunctival vitrectomy trocar system, which may offer combined 20-gauge fragmatome and 23-gauge vitrectomy for the management of denser lens nucleus in such eyes [28].

## Conclusion

In conclusion, our findings showed that the 23-gauge transconjunctival PPV combined with PPL was relatively safe and effective in the management of patients with glaucoma, extremely shallow anterior chamber and cataract. Definite anterior chamber formation and IOP reduction could be achieved by the combined surgical approach, with preservation of residual vision and decrease of anti-glaucoma medications. The procedure could be considered as a therapeutic option for eyes with poor visual function and no plan of IOL implantation.

## Abbreviations

BCVA: best corrected visual acuity
IOP: intraocular pressure
ACD: anterior chamber depth
PPV: pars plana vitrectomy
MIVS: microincision vitrectomy surgery
logMAR: logarithm of the minimum angle of resolution
LP: light perception
HM: hand movements
CF: counting fingers
IOL: intraocular lens
PPL: pars plana lensectomy
